# Performance evaluation of the LumiraDx quantitative microfluidic point-of-care CRP test

**DOI:** 10.1016/j.plabm.2023.e00349

**Published:** 2023-12-12

**Authors:** Jayne Ellis, James Harnett, Gregor Cameron, Phil Moss, Alasdair Gray

**Affiliations:** a JEMMDx Limited, Bedford, UK; b University College London Hospitals NHS Foundation Trust, London, UK; c ED Clinical Research Unit, St George's Hospital, London, UK; d Emergency Medicine Research Group (EMERGE), Royal Infirmary of Edinburgh, Edinburgh, UK

**Keywords:** C-reactive protein, CRP, Lower respiratory tract infection, Point-of-care test, Primary care, Secondary care

## Abstract

C-reactive protein (CRP) is an established acute-phase marker for infection, inflammation and tissue injury, used to guide clinical decision-making in primary and secondary care. This study compared the analytical performance of the quantitative microfluidic point-of-care LumiraDx CRP Test to a laboratory-based reference method (Siemens RCRP Flex assay on the Dimension® Xpand®) and evaluated equivalence of sample matrices (blood versus plasma) in point-of-care settings using samples from patients presenting with symptoms of infection or inflammation. The LumiraDx CRP Test demonstrated close agreement with the lab reference test (range, 5.1 to 245.2 mg/L, r = 0.992, slope = 0.998, intercept = –0.476; n = 205) and notable agreement between fingerstick and venous blood and plasma (r = 0.974–0.983; n = 44). Paired replicate precision had mean coefficients of variation of 6.4 % (plasma), 6.6 % (capillary direct) and 8.1 % (venous blood); overall error rates were 2.9 %. The quantitative LumiraDx CRP Test showed robust analytical performance across sample matrices and close agreement compared to the laboratory reference method when used at the point of care.

## Highlights

• C-reactive protein (CRP) is used as biomarker for infection and inflammation.
• It is used to guide clinical decision-making in primary and secondary care.
• The point of care LumiraDx CRP Test has demonstrated good analytical performance with plasma.
• It has also showed matrix equivalence between capillary blood, venous blood, and plasma.

## 1 Introduction

C-reactive protein (CRP) is an established clinical marker; its concentration in the circulation rises in response to infection and non-infectious inflammation, following increased secretion of inflammatory cytokines, particularly interleukin 6, by macrophages and T cells [1]. Conditions that affect CRP levels include rheumatoid arthritis, several cardiovascular diseases and infection [1,2]. The average level of CRP in the serum of a healthy Caucasian individual is approximately 0.8 mg/L [1,3], and it is generally accepted that levels greater than 10 mg/L indicate inflammation or infection; marked elevation of more than 100 mg/L can signify acute bacterial and/or viral infection or major trauma [4]. In the emergency department, CRP is measured in patients presenting with a variety of symptoms to aid detection and evaluation of infection, tissue injury or inflammatory disorders [5], and to guide antibiotic treatment decisions and therapy adjustment [[5], [6], [7], [8]]. In primary care, point-of-care (POC) CRP analysis is recommended when diagnosis is uncertain in suspected pneumonia (lower respiratory infections), where a low level of CRP (<5 mg/L) indicates that antibiotic therapy should be withheld as the infection is likely of a viral or a bacterial self-limiting nature [7,9,10]. Obtaining quantitative CRP results quickly from fingerstick blood samples is an important clinical innovation that can lead to pathway improvements. It is therefore important that POC CRP tests are robustly evaluated to ensure results are aligned with those from the central laboratory.

This study aimed to assess the analytical performance of the LumiraDx CRP Test, a microfluidic immunoassay for use in POC settings, when compared to a reference method, in patients with symptoms of infection, tissue injury or inflammatory disorders.

## 2 Methods

To evaluate the accuracy of the LumiraDx CRP Test, a comparison was made against a reference assay (Siemens RCRP Flex assay on the Dimension® Xpand®) using samples from the NOVEL prospective study, which enrolled patients (≥18 years) with symptoms of infection, tissue injury or inflammatory disorders. Lithium heparin-anticoagulated whole blood samples were processed to plasma for duplicate testing using the LumiraDx CRP Test and the reference test. The NOVEL study complied with the Declaration of Helsinki (2013) and was approved by the West of Scotland Research Ethics Committee 3 (REC number 15/WS/0176; System ID: 179093).

The precision and matrix equivalence of the LumiraDx CRP Test device was evaluated in patients (>=18 years) presenting with symptoms of infection, tissue injury or inflammatory disorders (REACT study [NCT05180110]). Venous blood (VB) was collected from each participant and tested immediately using the LumiraDx CRP Test; the remainder of the sample was processed to plasma, frozen and tested at the sponsor site (LumiraDx UK Ltd, Stirling, UK). Capillary blood samples were analysed at the point of care according to instructions [11]; two fingerstick samples (20 μL each) were applied via direct application (DA), and two fingerstick samples (20 μL each) applied to the test strip using a transfer tube (TT). The mean paired replicate precision of the POC CRP assay was also evaluated in this study; data were presented as the mean percentage of the coefficient of variation (%CV). The REACT study received approval from the South-East Scotland Research Ethics Committee (REC 19/SS/0115) and the Health Research Authority. The study protocol (REC 19/SS/0115) complied with the Declaration of Helsinki (2013).

Written informed consent from all participants of both studies was obtained prior to enrolment. The number and type of error codes observed with the LumiraDx test device were recorded for both studies. The haematocrit (HCT) range for the LumiraDx CRP Test device was 25–55 %. Samples with HCT outside this range were listed as an error and included in error rate analysis. Required sample sizes were determined to align with Clinical and Laboratory Standards Institute guidelines [10,12]. To assess method equivalency, a Passing–Bablok regression analysis was performed with pre-specified criteria of r≥0.95 and a slope of 0.95–1.05. Matrix equivalency of different testing modalities was assessed using Passing–Bablok regression, with the mean of two replicates analysed across all measured sample types (DA, TT, VB and plasma). To demonstrate equivalence of methods, all confidence intervals of the slope must contain 1.0 [13]. The overall study error rate was calculated by analysing all the raw data collected as part of both studies.

## 3 Results

### 3.1 Method comparison analysis

**Fig. 1:**
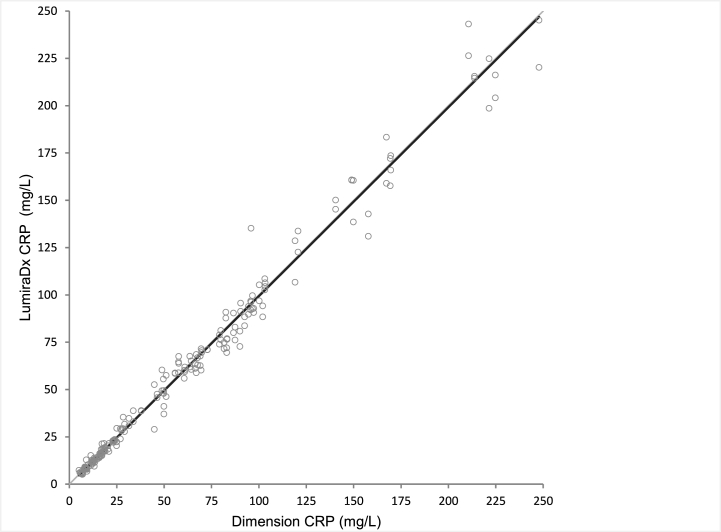
The Passing–Bablok regression analysis of the LumiraDx CRP plasma test results versus Siemens RCRP Dimension® Xpand® Plus plasma test.

A total of 205 VB samples from the NOVEL study were collected from 129 participants, age ranging from 20 to 96 years (median 58; mean 54), 48 % of whom were female. Plasma samples across the CRP measuring range of 5.1–245.5 mg/L were included and two test strip lots used on the LumiraDx CRP Test. Data analysis demonstrated close agreement of the LumiraDx CRP Test with the laboratory-based reference method, meeting the pre-specified performance criteria (r≥95 and a slope of 0.95–1.05) with r = 0.992, a slope of 0.998, and an intercept of –0.476. The Passing– Bablok analysis showed the requirement of r ≥0.95 was met (Table 1; Fig. 1).

**Table 1:** The Passing–Bablok regression results displayed as LumiraDx CRP plasma test results versus Siemens RCRP Dimension® Xpand® Plus Integrated Chemistry System plasma test results.

Strip lot	N	CRP range (mg/L)	Slope	Intercept	r^2^	r
All lots	205	5.1–245.2	0.998	–0.476	0.984	0.992
1	102	5.1–226.4	0.980	–0.067	0.980	0.990
2	103	5.1–245.2	1.008	–0.717	0.988	0.994

CRP, C-reactive protein; r, correlation coefficient; r^2^, correlation coefficient squared.

### 3.2 Paired replicate precision and matrix equivalency

**Fig. 2:**
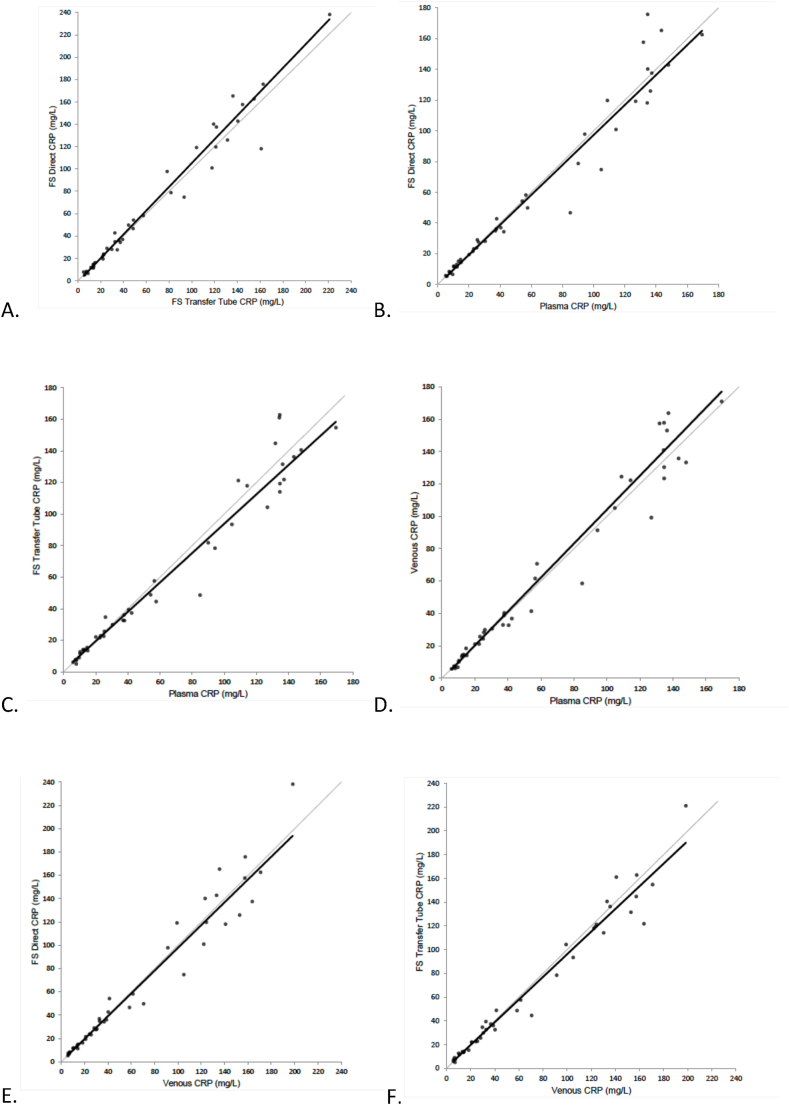
The Passing–Bablok regression analysis of matrix equivalency of the LumiraDx CRP when comparing different sample types. A: fingerstick (FS) transfer tube (TT) vs fingerstick direct application (DA); B: plasma vs fingerstick direct application (DA); C: plasma vs fingerstick transfer tube (TT); D: plasma vs venous blood; E: venous blood vs fingerstick direct application (DA); F: venous blood vs fingerstick transfer tube (TT). Abbreviations: %CV, coefficient of variation; CRP, C-reactive protein; DA, direct application; FS, fingerstick; HCT, haematocrit; POC, point-of-care; VB, venous blood.

Paired replicate precision and matrix equivalency analysis was conducted by POC operators on 44 participants with complete sample sets, where fingerstick blood (DA and TT), VB and plasma results were compared in duplicate. The age range of participants was from 18 to 84 years (median 55; mean 54) and 63 % were female. Paired replicate precision was assessed across a CRP measuring range of 19.9–185.4 mg/L for each sample type. The mean %CV for the three sample types was 6.4 for plasma, 6.6 for capillary DA, 7.6 for capillary blood TT and 8.1 for VB. Matrix equivalency analysis for the different test modalities using Passing–Bablok regression resulted in r values of 0.97–0.98 across all sample types, demonstrating matrix equivalency for all pairs (Table 2; Fig. 2).

**Table 2:** Passing–Bablok regression analysis of matrix equivalency for the different testing modalities.

LumiraDx sample type	N	CRP range (mg/L)	Slope	Intercept	r
Plasma versus venous blood	43	5.2–69.6	1.05	–0.79	0.981
Plasma versus capillary blood TT	44	5.2–169.6	0.93	0.90	0.977
Plasma versus capillary blood DA	44	5.2–169.6	0.98	–0.29	0.974
Venous versus capillary blood TT	44	5.7–198.5	0.95	0.73	0.983
Venous versus capillary blood DA	43	5.7–198.5	0.98	0.09	0.974
Capillary blood TT versus DA	44	5.1–221.2	1.06	–1.21	0.982

CRP, C-reactive protein*;* DA, direct application*;* r, correlation coefficient*;* TT, transfer tube.

### 3.3 Error rate analysis

The error rate analysis revealed an overall study error rate of 2.9 %. This included user as well as system error traps. Of the 31 test strip errors, 29 resulted from venous and capillary blood testing. The most common error in the REACT study was recorded as ‘Door close timeout’ (n = 9). This user error was corrected with product training. The second most common error (n = 7) resulted from unsuitability of samples owing to the HCT parameters being out of range.

## 4 Discussion

This study evaluated the analytical performance of a new CRP microfluidic immunoassay on the LumiraDx Test Platform in comparison to the Siemens RCRP Flex assay on the Dimension® Xpand® laboratory test system. The performance evaluation demonstrated notable equivalence between the two methods in plasma samples from patients with symptoms of infection, tissue injury and inflammatory disorders. The accuracy of the LumiraDx CRP Test was established across a range of CRP concentrations. Furthermore, the LumiraDx CRP results were consistent across capillary fingerstick samples, venous whole blood and plasma, and high precision was shown across the different sample types. Overall, the LumiraDx CRP Test showed a low error rate of 2.9 %. This indicates that the LumiraDx CRP test could replace current standard laboratory CRP tests where laboratory facilities are not easily available, and results are required quickly to facilitate clinical decision-making. Use of a portable, connected POC CRP test may offer significant pathway benefits for primary and secondary care where it can deliver accurate, laboratory-equivalent results in minutes [[5], [6], [7],^9^,[14], [15], [16]].

The limitations of this study include the use of two different patient cohorts when performing the method comparison and matrix equivalency analyses, which was due to restrictions resulting from the coronavirus disease 2019 pandemic. Usability of the LumiraDx CRP Test at primary care sites was not evaluated in this study. However, the LumiraDx Platform has previously been evaluated on the basis of use of D-dimer tests at primary care sites, in which capillary blood, VB and plasma were also sample types [17].

In conclusion, the LumiraDx CRP Test was determined to be an accurate alternative to the established laboratory-based reference method and provides the benefit of delivering a quantitative result from a fingerstick sample in 4 min at the point of care.

## Funding

Sponsorship for this study and Rapid Service Fee were funded by LumiraDx.

## Authorship

All named authors meet the International Committee of Medical Journal Editors criteria

for authorship for this article, take responsibility for the integrity of the work as a whole, and have given their approval for this version to be published.

## Author contributions

Jayne Ellis was responsible for the concept and design of the study, as well as drafting the manuscript. Jayne Ellis, James Harnett, Gregor Cameron, Phil Moss and Alasdair Gray all contributed to the generation and evaluation of the clinical data, manuscript preparation and critically reviewed the manuscript.

## Medical writing, editorial and other assistance

The authors acknowledge Anne-Marie Quirke and Kathrin Schulze-Schweifing, of integrated medhealth communication (imc), for medical writing support.

## Compliance with ethics guidelines

The NOVEL study complied with the Declaration of Helsinki (2013) and was approved by West of Scotland Research Ethics Committee 3 (REC number 15/WS/0176 and Integrated Research Application System ID: 179093). The REACT study received approval from the South-East Scotland Research Ethics Committee (REC 19/SS/0115) and the Health Research Authority. The study protocol (REC 19/SS/0115) complied with the Declaration of Helsinki (2013). All participants provided informed consent prior to participation.

## Thanking patient participants

The authors would like to extend their gratitude to the study participants for their involvement in the NOVEL and REACT studies.

## Declaration of competing interest

The authors declare the following financial interests/personal relationships which may be considered as potential competing interests: Jayne Ellis was a consultant to LumiraDx in the development of this manuscript.

## Data Availability

Data will be made available on request.

## References

[1] Sproston N.R., El Mohtadi M., Slevin M., Gilmore W., Ashworth J.J. (2018). The effect of C-reactive protein isoforms on nitric oxide production by U937 monocytes/macrophages. Front. Immunol..

[2] Volanakis J.E. (2001). Human C-reactive protein: expression, structure, and function. Mol. Immunol..

[3] Ma Wörns, Victor A., Galle P.R., Höhler T. (2006). Genetic and environmental contributions to plasma C-reactive protein and interleukin-6 levels--a study in twins. Gene Immun..

[4] Nehring S., Goyal A., Bansal P., Patel B. (2021). https://www.ncbi.nlm.nih.gov/books/NBK441843/.

[5] Su Y.-J. (2014). The value of C-reactive protein in emergency medicine. J Acute Dis.

[6] Cooke J., Llor C., Hopstaken R., Dryden M., Butler C. (2020). Respiratory tract infections (RTIs) in primary care: narrative review of C reactive protein (CRP) point-of-care testing (POCT) and antibacterial use in patients who present with symptoms of RTI. BMJ Open Respir Res.

[7] Primary Care Respiratory Society The place of point of care testing for c-reactive protein in the community care of respiratory tract infections 2022. https://www.pcrs-uk.org/crp-point-care-testing.

[8] Yardley L., Douglas E., Anthierens S. (2013). Evaluation of a web-based intervention to reduce antibiotic prescribing for LRTI in six European countries: quantitative process analysis of the GRACE/INTRO randomised controlled trial. Implement. Sci..

[9] NICE Clinical Guideline 191: pneumonia in adults: diagnosis and management. https://www.nice.org.uk/guidance/cg191.

[10] (2022). CLSI. EP28 Defining, Establishing, and Verifying Reference Intervals in the Clinical Laboratory.

[11] LumiraDx CRP test strip product insert 2022. https://www.lumiradx.com/assets/pdfs/crp-test/crp-product-insert/crp-test-strip-product-insert-ce-english-netherlands.pdf?v=3.

[12] CLSI EP09 measurement procedure comparison and bias estimation using patient samples. https://clsi.org/media/2293/ep09ed3ce_sample.pdf.

[13] Bilić-Zulle L. (2011). Comparison of methods: passing and Bablok regression. Biochem. Med..

[14] Butler C.C., Gillespie D., White P. (2019). C-reactive protein testing to guide antibiotic prescribing for COPD exacerbations. N. Engl. J. Med..

[15] Millot G., Voisin B., Loiez C., Wallet F., Nseir S. (2017). The next generation of rapid point-of-care testing identification tools for ventilator-associated pneumonia. Ann. Transl. Med..

[16] Nijman R.G., Moll H.A., Vergouwe Y., de Rijke Y.B., Oostenbrink R. (2015). C-reactive protein bedside testing in febrile children lowers length of stay at the emergency department. Pediatr. Emerg. Care.

[17] Ellis J.E., Johnston T.W., Craig D. (2021). Performance evaluation of the quantitative point-of- care LumiraDx D-dimer test. Cardiol Ther.

